# Conditional female strategies influence hatching success in a communally nesting iguana

**DOI:** 10.1002/ece3.6139

**Published:** 2020-03-04

**Authors:** Jeanette B. Moss, Glenn P. Gerber, Tanja Laaser, Matthias Goetz, TayVanis Oyog, Mark E. Welch

**Affiliations:** ^1^ Biological Sciences Department Mississippi State University Mississippi State MS USA; ^2^ Institute for Conservation Research San Diego Zoo Global Escondido CA USA; ^3^ Cayman Islands Department of Environment George Town Cayman Islands; ^4^ Department of Herpetology Durrell Wildlife Conservation Trust Jersey UK

**Keywords:** aggregative oviposition, early incubation environment, egg chamber, reproductive tactics, squamate reptile, West Indian rock iguana

## Abstract

The decision of females to nest communally has important consequences for reproductive success. While often associated with reduced energetic expenditure, conspecific aggregations also expose females and offspring to conspecific aggression, exploitation, and infanticide. Intrasexual competition pressures are expected to favor the evolution of conditional strategies, which could be based on simple decision rules (i.e., availability of nesting sites and synchronicity with conspecifics) or on a focal individual's condition or status (i.e., body size). Oviparous reptiles that reproduce seasonally and provide limited to no postnatal care provide ideal systems for disentangling social factors that influence different female reproductive tactics from those present in offspring‐rearing environments. In this study, we investigated whether nesting strategies in a West Indian rock iguana, *Cyclura nubila caymanensis*, vary conditionally with reproductive timing or body size, and evaluated consequences for nesting success. Nesting surveys were conducted on Little Cayman, Cayman Islands, British West Indies for four consecutive years. Use of high‐density nesting sites was increasingly favored up to seasonal nesting activity peaks, after which nesting was generally restricted to low‐density nesting areas. Although larger females were not more likely than smaller females to nest in high‐density areas, larger females nested earlier and gained access to priority oviposition sites. Smaller females constructed nests later in the season, apparently foregoing investment in extended nest defense. Late‐season nests were also constructed at shallower depths and exhibited shorter incubation periods. While nest depth and incubation length had significant effects on reproductive outcomes, so did local nest densities. Higher densities were associated with significant declines in hatching success, with up to 20% of egg‐filled nests experiencing later intrusion by a conspecific. Despite these risks, nests in high‐density areas were significantly more successful than elsewhere due to the benefits of greater chamber depths and longer incubation times. These results imply that communal nest sites convey honest signals of habitat quality, but that gaining and defending priority oviposition sites requires competitive ability.

## Graphical abstract

In this study, we investigated whether nesting strategies in the Sister Islands rock iguana, *Cyclura nubila caymanensis*, vary conditionally with individual competitive ability or reproductive timing, and evaluated consequences for nesting success. Reproductive strategies varied among females of different age, and success was shown to be dependent on local nest densities and the duration and depth of incubation within communal nest sites. Because individual conditions are likely to change over the lifetime, plasticity should be advantageous.

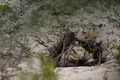

## 1 INTRODUCTION

Successful reproduction is central to fitness, and thus, understanding the causes and consequences of individual variation in reproductive strategies is a major goal of behavioral ecology. One such strategy is the tendency of gravid females to aggregate with conspecifics around the time of parturition or egg laying. While animals that reproduce together are often more likely to adopt cooperative strategies for rearing young (Kokko, Johnstone, & Clutton‐Brock, 2001; Riehl, 2013), communal aggregations also arise in “asocial” and territorial animals. Indeed, the same ecological factors thought to promote the evolution of alloparental brood care in birds (i.e., habitat saturation) are also implicated in the evolution of high‐conflict societies (e.g., conspecific brood parasites; Zink & Lyon, 2015), clarifying that the adaptive fitness benefits of aggregation need not be mutualistic. Individual‐level benefits include reduced energetic costs (e.g., associated with nest site selection: Brown & Shine, 2005; Radder & Shine, 2007; nest construction: Rand & Dugan, 1983; and brood care: Manning, Dewsbury, Wakeland, & Potts, 1995), diluted predation risks (Ims, 1990a), and thermoregulatory benefits (Williams et al., 2013). Conversely, communal settings elevate socially induced stress (Hill, Pillay, & Schradin, 2015) and introduce costs, including competition for space and resources (Graves & Duvall, 1995; Trumbo & Fiore, 1994), exploitation (Ferrari, Lindholm, & König, 2015; Lyon & Eadie, 2008), and infanticide (Cheetham, Doody, Stewart, & Harlow, 2011; Schmidt et al., 2015). In fact, competition among females appears to be a major driver of reproductive synchrony—a recurrent pattern associated with group formation across diverse taxa (reviewed in Ims, 1990b). This temporal clustering of reproductive events is consistent with stabilizing selection, as early parturition elevates the risk of infanticide by other gravid females, while late parturition leaves offspring vulnerable to starvation in competitions with older littermates (Ebensperger, Hurtado, & León, 2007; Hodge, Bell, & Cant, 2011; Poikonen, Koskela, Mappes, & Mills, 2008; Riehl, 2016).

Female–female competition within communal aggregations could also serve as a selective filter, promoting adoption of alternative strategies by a fraction of the population. Indeed, pronounced within‐sex competition and variance in reproductive success are expected to favor the evolution of different reproductive tactics (Brockmann, Grafen, & Dawkins, 1979; Hill et al., 2015; Taborsky, Oliveira, & Brockmann, 2008). These tactics may arise from genetically fixed differences between females or alternative strategies; but more often conditional strategies result from evolutionarily stable variation maintained by probabilistic decision rules (i.e., availability of breeding territories and synchronicity with other nesters), or decisions linked to individual condition or status (i.e., body size or compeitive ability; Hill et al., 2015; Shuster & Wade, 2003; Taborsky, 1998). That communal strategies almost always coexist with solitary strategies in populations where they have been studied (Doody, Freedberg, & Keogh, 2009; Weidt, Lindholm, & König, 2014) supports the notion that different tactics are favored under different circumstances. However, the causes and consequences of these individual‐level decisions have primarily been examined in mammals (Ebensperger et al., 2007; Ferrari et al., 2015; Manning et al., 1995; Schmidt et al., 2015) and birds (Lyon & Eadie, 2008; Vehrencamp, 2000; Vehrencamp, Koford, & Bowen, 1988; Zink & Lyon, 2015), for which the social factors influencing female nesting strategies may be impossible to disentangle from those present in offspring‐rearing environments. To understand more general principles that govern the coevolution of conditional female reproductive strategies, an expanded taxonomic scope (including species with limited to no postnatal parental care) is needed.

In oviparous, nonavian reptiles, offspring are autonomous at hatching, and while many species exhibit egg‐attendance behaviors, postnatal parental care is rare (reviewed in Doody, Burghardt, & Dinets, 2013). As a result, selection on nesting strategies is highly concentrated at the time of nesting, and many trade‐offs can occur between mothers and offspring (Mousseau & Fox, 1998; Refsnider & Janzen, 2010). Because hatchling phenotypes are highly labile to environmental conditions experienced during incubation (reviewed in Booth, 2006), strategies governing oviposition site selection (Wood & Bjorndal, 2000) and nest construction have received considerable attention in this group. Most egg‐laying reptiles oviposit in the ground, with some lizards capable of digging to extreme depths (reviewed in Doody et al., 2014). This buffers eggs against desiccation (Doody, James, Colyvas, Mchenry, & Clulow, 2015; Nelson, Thompson, Pledger, Keall, & Daugherty, 2004) and can prolong embryogenesis (Andrews, Pezaro, Doody, Guarino, & Green, 2017; Martins et al., 2007) to the effect of producing larger, more competitive offspring (Brown & Shine, 2006). However, the energy females expend assessing microhabitat quality and excavating nests can be considerable (Hayes, Carter, Cyril, & Thornton, 2004; Iverson, Hines, & Valiulis, 2004; Nelson et al., 2004; Rand, 1968). One strategy proposed to mitigate these costs is communal egg‐laying—a phylogenetically widespread trait in reptiles (Doody et al., 2009; Graves & Duvall, 1995; Radder & Shine, 2007). Experimental work has demonstrated that ovipositing lizards use conspecific eggs and eggshells as attractive cues for the placement of their own eggs (Brown & Shine, 2005; Radder & Shine, 2007), supporting the notion that habitat saturation alone does not explain clumped nest distributions (Doody et al., 2009; Graves & Duvall, 1995). Contrasting these potential benefits, observational data from lizards also portray communal nesting sites as highly competitive environments, with females engaging in intense physical confrontations over preferred sites (Iverson et al., 2004; Wiewandt, 1982; Wilson et al., 2016) and exploiting earlier nests, which often results in ovicide (Bock & Rand, 1989; Cheetham et al., 2011; Graves & Duvall, 1995; Rand & Dugan, 1983; Wiewandt, 1982). In addition, clumped egg distributions attract common nest predators (Marchand & Litvaitis, 2004). Thus, decision rules and individual condition likely factor importantly into communal nesting decisions and their outcomes.

A promising system for investigating these dynamics is the West Indian rock iguana (genus *Cyclura*), an insular lizard for which a range of nesting behaviors, including aggregative oviposition, have been documented (Carreras‐De León et al., 2019; Iverson et al., 2004; Pérez‐Buitrago, Sabat, & McMillan, 2016; Wiewandt, 1977). Much of the conversation surrounding communal nesting in *Cyclura* has focused on the constraining habitat features of Caribbean islands: predominantly karst limestone, with spurious distributions of deep, friable soil, access to which often demands long‐distance nesting migrations (Iverson et al., 2004; Moss et al., 2020; Pérez‐Buitrago et al., 2016). However, there is also considerable evidence that social factors (i.e., competition) could account for individual variation in nesting strategies. Rock iguanas inhabit tropical and subtropical latitudes with distinct wet and dry seasons, such that reproduction is highly seasonally concentrated (Iverson et al., 2004; Knapp, Iverson, & Owens, 2006; Pérez‐Buitrago et al., 2016; Wiewandt, 1977). As a result, interactions among nesting females are frequent, particularly within large aggregations. Because energetic considerations appear to be among the most important factors dictating reproductive decisions in iguanas (Rand & Rand, 1976, 1978), optimal nesting strategies should simultaneously minimize investments in nest construction while also avoiding or mitigating costly disputes. While rock iguana nesting seasons are typically highly punctuated (Iverson et al., 2004; Knapp et al., 2006; Pérez‐Buitrago et al., 2016; Wiewandt, 1977), females can spend a substantial portion of them defending nests against conspecifics (Iverson et al., 2004; Knapp & Owens, 2008; Wiewandt, 1977; Wilson et al., 2016). To minimize these costs while still gaining access to high‐quality oviposition sites, synchronicity with other nesters in an aggregation may be critical (Bock & Rand, 1989; Wiewandt, 1982, 1993). Individuals may also express different conditional strategies across their lifetime depending on their competitive ability in a given year. This is especially true of lizards including iguanas, for which the outcomes of competitions are correlated with body size (Alberts, Lemm, Perry, Morici, & Phillips, 2002; Stuart‐Smith, Swain, & Wapstra, 2007; Tokarz, 1985; Wegener, Mulder, Pringle, Losos, & Kolbe, 2019) and for which there exists substantial size variation among individuals due to indeterminant growth (Engqvist & Taborsky, 2015).

In this study, we report on four years of nesting surveys of free‐ranging *Cyclura nubila caymanensis* on Little Cayman, Cayman Islands, British West Indies (BWI). Our purpose was to investigate whether nesting strategies were predictable based on within‐season decision rules and/or individual condition, and to evaluate how social factors influenced reproductive success. We hypothesized that a female's preferred degree of spatial clustering would depend on seasonal reproductive timing and body size. Specifically, we anticipated that preference for lower‐density sites should be expressed early and late in the season due to exploitation avoidance and competitive exclusion, respectively, and that the smallest females would be disproportionately excluded from high‐density sites. In addition to aspects of the physical incubation environment, we hypothesized that hatching success would be sensitive to surrounding nest densities and to interactions with female phenology and size. Specifically, we predicted that within high‐density areas, the earliest nests and those dug by smaller females would be more susceptible to ovicide and therefore would exhibit lower clutch hatching success.

## 2 MATERIALS AND METHODS

### 2.1 Study system

Little Cayman has an area of 28.5 km^2^ and its nearest landmasses are Cayman Brac (7.5 km southeast), a small island supporting the only other natural population of *C. nubila caymanensis*, and Grand Cayman (100 km southwest), inhabited by *C. lewisi*. Habitat considered optimal for iguana nesting is sparse on Little Cayman. A comprehensive survey of coastal shrubland undertaken in 2010 (Goetz, 2010) identified five major communal sites on the island, with the majority of activity concentrated on the coastal west end. Nests were also identified along the southern coastline and at a mound of phosphate in the west end's interior. In 2012, the Little Cayman District of the National Trust for the Cayman Islands purchased a 1.12‐ha plot of land encompassing most of Preston Bay, the island's largest known communal nesting site. The purchase protected the site in perpetuity; however, monitoring of iguana nests was not resumed following 2010 surveys until 2015. In addition to recording solitary nests opportunistically, we selected eight communal nesting localities on the west end (2015–2018) and three on the southern coastline (2017) for regular monitoring in this study (Appendices S1 and S2).

### 2.2 Nest surveys

Surveys of nest sites took place over two time intervals—the nesting season (May–June) and the emergence season (August–September). Survey sites were visited twice daily (with the exception of prohibitively inclement weather; Appendix S3), to note developments associated with nesting (detailed by Iverson et al., 2004). Briefly, these included direct observation of digging females, the appearance of newly excavated entrance tunnels, and discolorations in the substrate concealing freshly filled entrance tunnels. Upon completion, nests were assigned unique IDs, marked with flagging tape, and georeferenced with a handheld GPS. Nests that were reopened and subsequently resealed by a female other than the nest's original constructer were documented independently. Twice daily surveys were resumed in early August to record emergence events. Emergence of a nest was inferred by the appearance of an “escape” hole above the egg chamber and confirmed via excavation of the egg chamber (detailed in *Nest excavations*, below). A nest's incubation length was recorded as the number of days between nest closure and emergence.

### 2.3 Individual capture and identification

Iguana captures were carried out by noose, net, or appropriately sized live traps (Tomahawk Live Traps) outside of nesting sites as well as within active nesting areas. To reduce disturbance to females during oviposition, captures were limited to periods after nest closure when females were spent. To differentiate between individual females and associate marked nests with nesters, iguanas were marked in two ways: externally with a unique color combination of small, glass beads secured through the nuchal crest using durable Nanofil fishing line, and permanently with HPT8 MiniChip Passive Integrated Transponder tags (BioMark) injected subdermally at the dorsal tail base. Standard morphometric measurements were collected for each animal, including snout‐vent length (SVL in mm) and body mass (in g).

**Figure 1:**
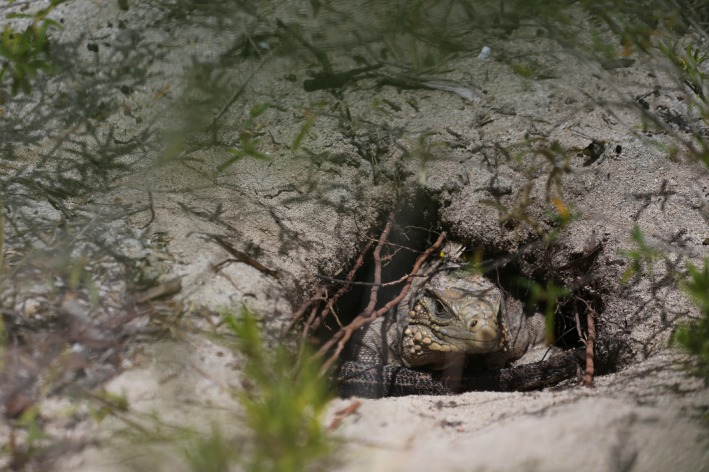
A gravid female *Cyclura nubila caymanensis* on Little Cayman during a rest from digging her nest. Photograph by Michael Kartje

When females were documented nesting in multiple years, these data were leveraged to evaluate the repeatability of individual site selection decisions. Because detection of females at nest sites was imperfect, we employed pedigree reconstruction of whole clutches of offspring (detailed by Moss, Gerber, Schwirian, Jackson, & Welch, 2019), as a secondary tool for identifying nesters among previously sampled candidates. Genetic samples were obtained from all animals captured, including hatchlings, in the form of small volumes of blood (0.5–1.0 ml) drawn from the ventral caudal vein. Minimum return rates of tagged nesters were estimated from year‐to‐year as well as at two‐ and three‐year intervals to account for iteroparity and/or failure of detection (Figure 1).

### 2.4 Nest excavations

Wherever possible, nests were excavated to investigate structure and evaluate hatching success (Appendix S4). Excavating iguana nests by hand is time and labor intensive, and not all nests that were identified within a nesting season could be excavated. However, many nests that were not excavated during the nesting season were excavated following hatchling emergence by digging out the emergence tunnel. These “postemergence” excavations facilitated determinations of clutch size, hatching success, and depth to the floor of the egg chamber for nests possessing limited nesting‐season data. Substrate types were recorded as sand, predominantly rock (“rocky”), rocky sand (a mixed substrate composed of both rocks and sand), or phosphate. For a sample of nests in 2016 (*N* = 15), HOBO Pendant data loggers (Onset) were deployed within egg chambers at the start of incubation and programmed to report temperature ±0.53°C every five minutes until nest emergence.

### 2.5 Statistical analyses

All statistical analyses were performed in R v. 3.5.0 (R Core Development Team, 2017). To quantify nest densities, a modified edge‐thinning technique (Brooks, 2006; Appendix S5; Figure S1) was employed to define three threshold distances of spatial clustering—85, 140, and 440 m (Figure S2)—and hierarchically partition nests into spatial “neighborhoods.” Based on the observation that female rock iguanas often search large areas and initiate many test digs before ultimately selecting a site to oviposit (Pérez‐Buitrago et al., 2016; Rand & Rand, 1976; J. B. Moss personal observation), we predict that nest clustering at these broad spatial scales will have important consequences for female behavior. To characterize variation in fine‐scale clustering, an additional threshold distance of 10 m was considered. Nest counts were obtained for each neighborhood by year, and nearest neighbor distances were calculated with the R package “spatstat” (Baddeley & Turner, 2005). Parameter estimates were restricted to nests within regularly monitored sites such that analyses would reflect all possible interactions among surveyed nests. Nest counts were found to be increasingly intercorrelated across successive spatial scales and were also highly correlated with nearest neighbor distances (Figure S3).

Dates of nest closure (start) and emergence (end) for each nest were defined in Julian days. To account for the possible role of climatic and social cues in regulating within‐season phenology, dates were scaled relative to each year's activity peak (estimated from daily nest counts). To reduce the dimensionality of our dataset and explain correlations among nest parameters using a smaller number of underlying factors, we employed multivariate principal component analysis (PCA). We accounted for incompleteness in our dataset by preparing a reduced sample (*N* = 120) consisting of observations with no more than one of the following parameters missing: chamber depth (*N* = 115), tunnel length (*N* = 98), incubation length (*N* = 100), neighborhood nest counts within 85, 140, and 440 m (*N* = 120), and nearest neighbor distance (*N* = 118). Outstanding missing values were estimated via nonparametric multiple imputation, as implemented in the R package “missMDA” (Josse & Husson, 2016). Nearest neighbor distances were log_10_‐transformed and inverted such that all neighborhood parameters would show the same directionality (higher values correspond to greater neighborhood nest counts and nearer neighbors). The prcomp function in R was used to perform the PCA. Principal components (PCs) were retained if they produced eigenvalues >1 and factor loadings with eigenvectors greater than 0.4 or <−0.4 were used to characterize each PC (Kaiser, 1960).

Mixed‐effects modeling approaches implemented in the R package “lme4” (Bates, Maechler, Bolker, & Walker, 2015) were employed to evaluate the interrelatedness of variables in our dataset. We tested for linear and quadratic responses of female body size (measured as SVL) and female nesting strategy (individual PC scores) to reproductive timing (relative start date). Female body size was also investigated as a possible predictor of nesting strategy. Finally, the influence of phenology, female body size, nesting strategy, and their interactions on clutch hatching success were examined via a series of binomial logistic regression models weighted by clutch size. Site, substrate, and year were specified as random effects in all models, and female ID was incorporated as a random effect in all models testing effects of female body size (i.e., for which female identity was known).

## 3 RESULTS

**Figure 2:**
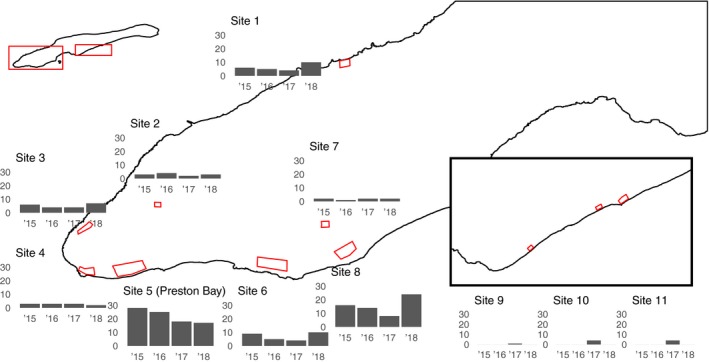
Annual nest counts at major sites. Sites 1–8 on the west end were monitored consistently between 2015 and 2018, while Sites 9–11 on the southern coastline were monitored only in 2017. Each site is described in detail in Appendix S2

We identified a total of 296 iguana nests on Little Cayman between 2015 and 2018. Of these, 259 occurred at one of the eight west end sites regularly monitored for all four years, and nine occurred at sites on the southern coastline that were monitored throughout the 2017 season (Figure 2). The remaining 28 nests were identified in island areas not regularly monitored by survey. Our overall estimates of local densities were low compared with those reported at the same sites in 2010 (Goetz, 2010); however, we acknowledge that much of this difference can be attributed to differences in survey methodologies (Appendix S1) and/or shifts in population dynamics. A summary of all parameter values collected from individual nests across all four years is provided in Table 1.

**Table 1:** Summary data for all nest attributes measured across all survey years

Parameter	*N*	Minimum	Maximum	Mean	Standard error
Phenology					
Julian start day	241	129	179	158.74	8.92
Julian end day	171	212	257	234.67	7.45
Maternal characters					
Female SVL (mm)	80	283	480	401.1	4.0
Female mass (g)	79	620	6,050	2,979.94	113.77
Clutch size	151	3	25	12.79	0.34
Average egg length	34	54.48	70.65	61.24	0.56
Average egg width	34	36.05	49.21	42.96	0.39
Average egg mass	35	44.18	78.21	61.80	1.25
Incubation conditions					
Incubation length (days)	134	58	88	75.71	0.54
Tunnel length (cm)	106	20	473	165.74	8.97
Chamber depth (cm)	145	10	80	39.90	1.22
Mean chamber temp (°C)	15	31.84	34.42	33.10	0.18
Mean temp fluctuations (°C)	15	0.14	2.82	0.61	0.18
Nest density					
85 m no. of nests	261	1	12	5.44	0.18
140 m no. of nests	261	1	18	6.98	0.27
440 m no. of nests	261	1	27	14.01	0.53
Nearest neighbor distance (m)	257	1	93.34	12.27	1.00
Nest success					
Pre‐emergence clutch success	38	0.44	1.00	0.96	0.02
Postemergence clutch success	146	0.00	1.00	0.89	0.02

Note

Phenology refers to the Julian day on which nests were closed (start), and when hatchlings emerged (end). Maternal characters include animal snout‐vent length (SVL), and mass in addition to clutch size, and egg attributes. Incubation conditions refer to attributes of nests. Nest density refers to the number of nests in neighborhoods of sizes defined by the edge‐thinning technique (see Section 2). Nest success refers to the proportion of viable offspring in nests. Pre‐emergence clutch success = the proportion of viable eggs observed following nest excavation in the initial 1–23 days of incubation. Postemergence clutch success = the proportion of hatched eggshells removed from an egg chamber following nest emergence at the end of incubation.

### 3.1 Nesting strategies

**Figure 3:**
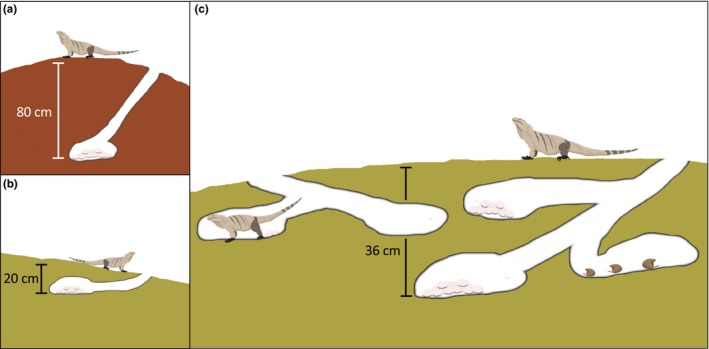
Cross‐sectional schematics of iguana nests on Little Cayman, illustrating approximate tunnel dimensions and egg chamber depths obtained from three representative excavations: (a) a nest dug in a deposit of phosphate (Site 2); (b) a shallow nest dug in sand (Site 1); and (c) neighboring nests at a large communal site (Site 5). Each has been re‐entered by conspecifics, resulting in multiple, branching egg chambers containing separate clutches. One chamber in the nest on the right contains eggs destroyed by another intruding female. Nests are not drawn precisely to scale

Female nest construction strategies as inferred via excavation were highly variable (Figure 3). While tunnel lengths varied widely, no significant effect of site (ANOVA: *F*_9,76_ = 1.8, *p = *.082) or substrate (ANOVA: *F*_3,82_ = 0.66, *p = *.594) was detected. However, tunnel length was positively correlated with egg chamber depth (*t* = 2.67, *R* ^2^ = .06, *p = *.009). Egg chambers were located significantly deeper in phosphate (Figure 3a) than in sand (Tukey's HSD test: *p = *.007; Figure 3b), rocky sand (Tukey's HSD test: *p = *.006), or rocky (Tukey's HSD test: *p = *.009) substrates. Mean daily temperature fluctuations (maximum daily temperature – minimum daily temperature), but not mean daily temperatures, were larger in shallow chambers (*t* = −5.66, *R* ^2^ = .69, *p* < .0001).

Local densities were highly variable within regularly monitored sites. The number of nests at the coarsest scale quantified for focal sites (440 m) ranged from one to 27 (Figure 2). Preston Bay (Site 5) supported the highest density of nests in each year except for in 2018, following a consistent decline in counts at the communal site between 2015 (*N* = 28) and 2018 (*N* = 17). We detected a total of 46 completed nests that shared some part of their tunnel with one or more neighboring active nests (Figure 3c). By this estimation, 7.3% of all egg‐filled nests and 20.3% of the egg‐filled nests at Preston Bay were subsequently dug into by one or more intruding gravid females. The majority of nests that shared tunnels possessed separate egg chambers. In at least one instance, two females were inferred via genetic reconstruction to have oviposited in the same egg chamber. Because genetic sampling of individuals was incomplete, the frequency of this behavior could be underestimated.

### 3.2 Phenological patterns

We observed activity seasons of five to seven weeks for both nesting and emergence (Figure S4). Pronounced peaks of between two (2018) and three (2015) weeks in duration encompassed 85% of nesting activity each year, and these consistently fell between the dates of 25 May and 17 June (2015 median: 09 June; 2016 median: 08 June; 2017 median: 07 June; and 2018 median: 11 June). Peak emergence dates fell between 12 August and 03 September each year (2015 median: 21 August; 2016 median: 21 August; 2017 median: 23 August; and 2018 median: 24‐August). Start dates did not vary significantly between survey years (ANOVA: *F*_3,84_ = 0.343, *p* = .794), but we detected significant pairwise differences in end dates between 2015 and 2018 (Tukey's HSD test: *p* < .0001) and between 2016 and 2018 (Tukey's HSD test: *p = *.002), likely due to staggered survey periods for the latter half of each season. Variance in start dates did not deviate from variance in end dates of paired nests (*F* test: *p = *.106). Inferred incubation lengths (2015 mean: 73.12 ± 7.21 days; 2016 mean: 75.43 ± 5.66 days; 2017 mean: 76.19 ± 5.43 days; and 2018 mean: 77.39 ± 5.92 days) were marginally left‐skewed (skewness test: s = −0.22) and were significantly shorter in 2015 than in 2018 (Tukey's HSD test: *p = *.010). One possible explanation for unusually short incubation lengths observed in our dataset (i.e., less than 65 days; *n* = 4) is that females sealed underground in nests for several days were not detected until their emergence, postlaying (Gerber, pers. comm.). If this was the case, then recorded nest starting dates would have been later than the start of incubation. However, because such instances are challenging to extricate from our dataset without introducing bias, all data analyses that follow utilize the complete dataset.

### 3.3 Female behaviors and reproductive output

A total of 81 unique tagged females were documented nesting on Little Cayman between 2015 and 2018. The smallest recorded gravid female in our dataset (28.3 cm in SVL and 1,080 g in mass) was inferred to be 2–3 years of age based on size trajectory analysis (Moss et al., 2020). Female SVL (2015 mean: 40.47 ± 4.35 cm; 2016 mean: 39.35 ± 3.04 cm; 2017 mean: 39.74 ± 3.85 cm; and 2018 mean: 42.19 ± 1.97 cm) was significantly positively correlated with clutch size (*t* = 3.41, *R* ^2^ = .199, *p = *.001; Figure S5). While individual egg dimensions did not vary with clutch size, eggs of smaller females were found to be significantly longer (*t* = −2.37, *R* ^2^ = .274, *p = *.031) and narrower (*t* = 4.71, *R* ^2^ = 0.597, *p = *.0003), which may reflect compensation for small body cavities (Iverson et al., 2004).

Among tagged females that were first documented in 2015 (*N* = 33), 82% were observed or genetically inferred to have returned to the same site to nest in a subsequent year (2016, 2017, and/or 2018; Table 2). Observed return rates were markedly reduced between 2017 and 2018; only 55% of 2016's nesters (*N* = 53) were observed nesting again, likely reflecting lower numbers of nesters recorded in these years. Females were documented nesting at a site other than their original recorded nest site on four occasions, and mortality explained at least four occasions when females failed to return in a subsequent year. Between‐year differences in nesting dates for females that were documented nesting in multiple years (*N* = 44) varied between one and 28 Julian days (mean = 7.05 ± 6.15 days).

**Table 2:** Number of females tagged at each survey site and return rates by year

Site	No. of nesters tagged/recorded (total no. of nests recorded)				Return rates			
	2015	2016	2017	2018	2015–2016 (%)	2015–later year (%)	2016–2017 (%)	2016–later year (%)
1	1 (6)	1 (5)	0 (4)	1 (10)	100	100	0	0
2	2 (3)	3 (4)	2 (2)	2 (3)	100	100	67.7	67.7
3	4 (6)	4 (4)	3 (4)	3 (7)	100	100	50	50
4	1 (3)	2 (3)	2 (3)	1 (2)	0	0	100	100
5	13 (28)	18 (25)	12 (18)	3 (17)	38.5	69.2	27.8	33.3
6	4 (9)	4 (5)	2 (4)	1 (10)	50	75	25	25
7	2 (2)	1 (1)	2 (2)	2 (2)	50	100	100	100
8	6 (16)	17 (14)	3 (8)	13 (24)	100	100	17.6	82.4
Overall	33	51	26	25	63.6	81.8	31.4	54.9

### 3.4 Interactions of nest parameters

**Figure 4:**
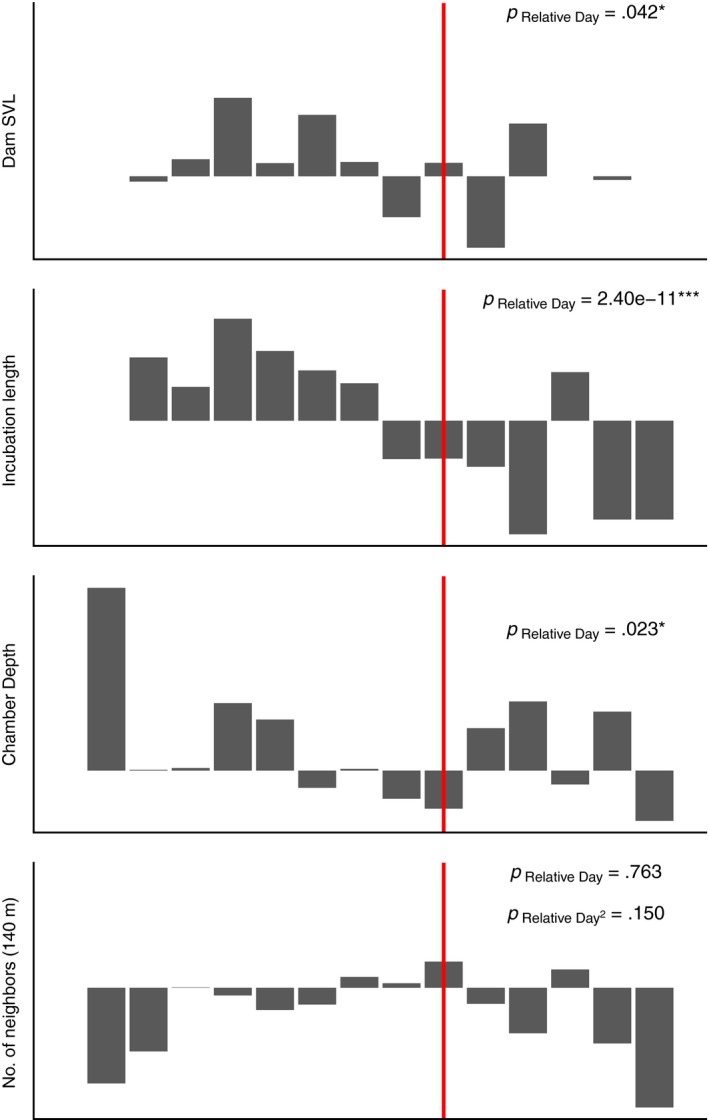
Temporal trends in female body size, incubation length, chamber depth, and number of neighbors (local densities) within 140 m for individual nests across the pooled range of nest survey dates. All parameters are displayed as Z scores and plotted against relative start dates. Red lines represent dates of peak nesting activity across pooled survey years. Observations are binned by intervals of four days and bar heights represent mean values of nests contained in the respective bin. *p*‐Values in each plot were obtained from linear mixed effect models of relative start date on each parameter, with site and year specified as random effects

Two principal components were retained from the PCA of nest incubation conditions, which together explained 57.6% of variance in strategy (Table 3). Neighborhood nest counts loaded highly and positively on PC1 (for convenience, the “local densities” axis), while deep chambers and long incubation periods loaded highly and positively on PC2 (the “incubation depth and duration” axis). Female body size was not significantly correlated with either PC1 or PC2. Female body size (likelihood ratio test: $\chi_{1}^{2}$ = 4.26, *p* = .042) and incubation depth and duration (likelihood ratio test: $\chi_{1}^{2}$ = 20.10, *p* < .0001) exhibited significant inverse linear relationships with relative start date. In other words, late‐season nests were generally constructed by smaller females, were dug to shallower depths, and exhibited shorter incubation periods (Figure 4; Figure S6). Quadratic responses of PC1 to relative start date were consistent with the maximum preference for high‐density sites coinciding with the phenological peak of the nesting season. However, this trend failed to reach statistical significance.

**Table 3:** Factor loadings from a multivariate principal component analysis of incubation condition parameters

Loadings	PC1	PC2
	39.80%	17.80%
Incubation length	−0.074	**0.637**
Tunnel length	−0.247	0.371
Chamber depth	−0.255	**0.509**
No. of nests (85 m)	**0.516**	0.173
No. of nests (140 m)	**0.536**	0.092
No. of nests (440 m)	**0.479**	0.030
Neighbor proximity	0.292	0.397

Note

Percentages indicate the amount of total variance explained by each axis. Two principal components with eigenvalues >1 were retained from this analysis. Factor loadings with eigenvectors >0.4 or <−0.4 are bolded.

### 3.5 Effects on clutch hatching success

Clutch hatching success was high overall (89.4 ± 21.6%; *N* = 146), including among nests that shared tunnels with other nests (88.3 ± 25.3%; *N* = 32). Inviable eggs were recovered from 39% of egg chambers following emergence, and 23.6% of egg failure was dated to within the first 1–23 days of incubation among nests excavated both within this window and again following emergence. Although high rates of nest re‐entry were observed among unguarded nests, we documented few instances of clutch disturbance or destruction resulting directly from antagonistic female behaviors. Hatching success below 65% attributable to environmental effects was observed in only 11.1% of excavated nests. A specific explanation for low hatching success (attack by ants) was identified in only three instances, while remaining cases were attributed to unknown causes (possibly maternal effects or disturbance by conspecifics). Failure to detect emergences in nests with clearly marked egg chambers (*N* = 13) may be attributed to total clutch failure. Total clutch failure was directly observed on three excavations, two of which involved females laying small clutches of entirely inviable eggs. The third occasion involved a nest that was re‐entered four times, and upon the fourth re‐entry, >30 inviable eggs were discovered in the main tunnel and in a terminal egg chamber. While direct evidence is lacking from this study, clutch failure resulting from disturbance by conspecifics may occur more frequently than we detected due to our limited capacity to excavate on multiple occasions.

Results of mixed‐effects models revealed significant independent contributions of relative start date and both nesting strategy PCs to clutch hatching success (Table 4). Hatching success increased with later relative start dates and greater incubation depth and duration but decreased with higher local densities. The interaction of incubation depth, duration, and local density was associated with significantly improved hatching success. This interactive effect was larger than that of either principal component alone, suggesting that elevated risks of egg damage with crowding can be more than offset by the construction of deep chambers with long incubation periods. While the strategies of aggregative nesting and deep nest construction exhibited different relationships with relative reproductive timing, we detected no significant effects on hatching success resulting from the interaction of either PC with relative start date or female body size.

**Table 4:** Results of binomial logistic regression models of clutch hatching success weighted by clutch size

Source of variation	Contribution to clutch hatching success			
	Estimate	*SE*	*F* (*df_n_*, *df*_d_)	Pr > *F*
Relative start date	0.024	0.011	4.367	.038*
Female body size	0.071	0.061	1.355	.240
Local densities	−0.186	0.088	4.557	.034*
Incubation depth and duration (PC2)	0.203	0.101	4.146	.045*
Local densities (PC1) × Incubation depth and duration (PC2)	0.198	0.065	9.185	.002**
Local densities (PC1) × Relative start date	−0.001	0.006	0.019	.891
Local densities (PC1) × Female body size	0.057	0.054	1.102	.291
Incubation depth and duration (PC2) × Relative start date	0.017	0.012	2.014	.156
Incubation depth and duration (PC2) × Female body size	−0.148	0.093	2.378	.113

Note

Significance values are reported for all fixed effects and their interactions. All models specify site and year as random effects. Models including dam body size as a fixed effect also specify residency status as a random effect. Significance levels (Pr > *F*) are indicated with *<.05 and **<.005.

## 4 DISCUSSION

We evaluated variation in individual nesting strategies across four years of survey effort in a free‐ranging rock iguana to characterize conditions that inform female reproductive decisions and determine reproductive success*.* Our observations of communal nest sites were consistent with studies portraying lizard aggregations as highly competitive. We documented exploitative nest excavation behaviors and significant hatching failure associated with high nest densities. While these high‐density sites did not appear to competitively exclude nesters based on body size, differently sized females exhibited different nesting phenologies. Specifically, an inverse relationship was observed between female body size and reproductive timing, suggesting that the strategy of early nesting is restricted to larger, potentially more competitive animals. There are a number of factors including the availability of resources for egg provisioning that could account for the later average parturition dates in smaller females. However, this finding is clearly consistent with the expectation that only females capable of investing in extended nest defense are able to overcome the risks of conspecific exploitation and ovicide early in the season. Furthermore, decreasing preference for high‐density areas following seasonal nesting peaks implies that competitive exclusion occurs later in the season. Variance in reproductive success was ultimately explained by a combination of variables, which included nest social environment but also encompassed other aspects of natal incubation environment. Nest depth, which was associated with greater thermostability and longer incubation times, positively correlated with hatching success. However, as the season progressed females appeared to forego these advantages in return for shallower nests and shorter incubation times. Possible advantages of later nesting strategies include that shallower nests require less energy to dig, and shorter incubation times may reduce exposure to incubation hazards such as ants. Combining communal strategies with favorable nest structural characteristics significantly increased hatching success, suggesting that communal nest sites possess properties important to the success of offspring as well as the fitness of females.

While comprehensive surveys of Little Cayman's coastline have identified large tracts of suitable habitat for iguana nesting (Goetz, 2010), our survey results are consistent with previous work documenting highly irregular nest densities. Sites ranged from supporting only solitary nests to aggregations of between 20 and 30 nests at Preston Bay, a 1.12‐ha communal site on the westernmost coastline. Our hypothesis was that variation in social environmental preferences among nesting females would be explained by differential energetic constraints linked to reproductive timing and body size. While female rock iguanas nesting in aggregations aggressively defend nests against conspecifics (Iverson et al., 2004; Knapp & Owens, 2008; Wiewandt, 1982; Wilson et al., 2016), energy available for nest defense may be reduced substantially following long‐distance nesting migrations (Iverson et al., 2004; Moss et al., 2020; Pérez‐Buitrago et al., 2016) and physical nest excavation. Evidence for this trade‐off is clear in the related *Iguana iguana*, for which females are visibly emaciated postlaying (Rand & Rand, 1976) and, having little energy to devote to nest defense prior to departing a nesting area, suffer many instances of conspecific intrusion and ovicide (Rand, 1968; Rand & Dugan, 1983; Rand & Rand, 1978). Rock iguanas appear to be exposed to these same risks, as we detected significant reductions in hatching success associated with high surrounding nest densities. We estimated that intruding females dug into approximately 20% of egg‐filled nests at Preston Bay, similar to conspecific intrusion rates of 10%–15% reported for a major communal nesting site on Mona Island (Wiewandt, 1977). On at least one occasion, we found that conspecific intrusion resulted in the total destruction of 1–2 large clutches of eggs. Observations of ovicide caused by conspecifics have also been made by Iverson et al. (2004), Hayes et al. (2004), and Knapp, Prince, and James (2016). Such anecdotal accounts likely represent only a portion of the fitness consequences of female–female competition within communal sites. Although many nests that were intruded upon still experienced high hatching success, even a small chance of total clutch failure should select strongly for behaviors that mitigate this risk. Overall, hatchling survivorship to emergence was high (0.89, Table 1), a finding slightly higher, yet consistent, with that estimated for *C. cychlura inoronata* (0.79, Iverson et al., 2004) and with that for *C. cychlura cychlura* (0.74, Knapp & Owens, 2008).

Our data provide some support for the hypothesis that female–female competition behaves as a reproductive synchronizer (Hodge et al., 2011; Poikonen et al., 2008; Wiewandt, 1993). While nests were detected at high‐density sites both early and late in the season, we observed that the strategy of communal nesting was increasingly favored up to the seasonal peak of nesting and appeared to diminish beyond this point. Several well‐studied *Cyclura* systems are similarly characterized by punctuated and highly predictable nesting seasons (Iverson et al., 2004; Knapp et al., 2006; Pérez‐Buitrago et al., 2016; Wiewandt, 1977), yet phenologies show no relationship with latitude and vary even among closely related taxa and geographically proximate populations (Iverson et al., 2004; Knapp et al., 2006). Rather, reluctance to invest in extended nest defense early in the nesting season, and competitive exclusion from communal sites toward the end of the nesting season may favor locally adapted phenologies between nesting communities. Contrary to expectations, however, we found no evidence that larger females, who should be better able to defend nests and usurp those of competitors, were more likely to nest communally. It is possible that young, first‐time nesters select communal sites by copying the choices of older conspecifics and gain nesting experience with minimal energy expenditure through the exploitation of existing tunnel networks. Further, small females might modulate costly interactions with larger conspecifics via timing of oviposition. Indeed, trends of decreasing female body size within sites as the nesting season progressed, similar to those documented in other *Cyclura* (Alberts, 1995; Iverson et al., 2004), implicate individual competitive ability as a precursor to investment in extended nest defense.

Reproductive success in our dataset was predicted by a number of variables, including aspects of natal incubation environment as determined by nest structural characteristics. Increasing nest depth appeared to buffer eggs against extreme daily temperature fluctuations, although unlike in other studies of reptiles (Doody et al., 2015; Packard & Packard, 1988), exhibited no correlations with mean daily chamber temperature. Hydric properties of egg chambers were not quantified in this study, but we suspect that desiccation may be more likely with less thermostability in shallow nests. In any case, incubation environments achieved at greater depths appeared to significantly improve hatching success. These benefits likely derive not only from reduced desiccation risk, but also from reduced risks of egg chamber collapse and predation. However, the exact cause of egg mortality could not be determined in most cases. Nest depth might also extend indirect benefits to offspring. Consistent with patterns previously described in other reptiles (Andrews et al., 2017; Martins et al., 2007), we detected a significant positive correlation between egg chamber depth and incubation time. Our measure of incubation time could not account for periods of latency between egg hatching and emergence, which may have been greater for deeper chambers due to the greater tunneling effort required for hatchlings to surface. Nonetheless, a nonmutually exclusive explanation for this phenomenon is that embryogenesis in reptiles proceeds more slowly at cooler, more stable temperatures (Du, Radder, Sun, & Shine, 2009; Du & Shine, 2010). Longer incubation has been shown to enhance offspring size and performance in reptiles by allowing for full absorption of yolk and possibly promoting further differentiation prior to emergence (Shine & Olsson, 2003). Consistent with this expectation, a companion study of *C. nubila caymanensis* (Moss, Gerber, & Welch, 2019) identified links between incubation duration and neonatal body size, a potentially important trait for locomotor performance and predator evasion.

These cofactors of reproductive success were also found to vary with reproductive timing. As the seasons progressed, nests were dug to shallower depths and incubation periods shortened, likely explaining temporal declines in nest performance (Table 4). It is possible that females nesting at the end of the season dug shallower nests due to constraints surrounding excavation. For example, late‐nesting females tended to be smaller and may not have been physically capable of excavating nests to extreme depths or were competitively excluded by other females, in which case they might have adopted “quick excavation” strategies to escape notice. Alternatively, digging shallower nests could represent a behavioral adjustment to reduce incubation duration. This would suggest strong selection on the timing of hatching. Indeed, despite year‐to‐year predictability in timing of nesting, considerable variation in incubation lengths indicates that this trait is labile and could be implicated in trade‐offs between time in development and timing of hatching. Evidence for this trade‐off exists in lizards, for which seasonally late hatching has been linked to reduced rates of juvenile growth and survival (Warner & Shine, 2007) and in the West Indies, drowning following storm‐associated rainfall (Knapp & Valeri, 2008, Iverson pers. comm., Gerber per. comm.). Because Little Cayman is a small island and scramble competition for food resources likely occurs at multiple ecosystem levels, we infer that earlier hatching should be favored to coincide with seasonal high resource availability (Shine & Olsson, 2003; Warner & Shine, 2007) and lower predator densities (Doody & Paull, 2013). Indeed, native snakes (*Cubophis ruttyi*) typically converge on Little Cayman's coastal nesting areas around the peak of hatching season (J. B. Moss personal observation), and late emerging hatchlings may disproportionately fall target to predators attracted to lingering nest site odors (Cheetham et al., 2011). Consistent with this expectation, rapid dispersal away from natal sites has been shown to improve early survival outlook in rock iguanas (Knapp, Alvarez‐Clare, & Perez‐Heydrich, 2010).

Finally, our study demonstrates that the interaction of nest structural and phenological attributes and surrounding social environment plays an important role in shaping reproductive outcomes. The expectation that communal nest sites introduce trade‐offs between the interests of mothers, such as presumed reduced energetic expenditure on excavation through exploitation of conspecific digging efforts, and offspring, such as increased risk of ovicide for early‐laid clutches by conspecific gravid females, was partially met in our study. Evidence for frequent re‐use of existing tunnel networks supports the hypothesis that females gain direct energetic benefits through communal nesting, which could explain high rates of between‐season nest site philopatry observed in this study and in females of related taxa (Iverson et al., 2004; Knapp & Owens, 2008; Pérez‐Buitrago et al., 2016). However, our second hypothesis was not supported. We found that individual nest phenology within dense aggregations and rates of hatching failure were not correlated. Hence, while high nest densities expose eggs to an increased probability of ovicide by intruding conspecifics or density‐dependent hatchling depredation, our data suggest that these costs are outweighed by the benefits of nesting within the high‐density sites. Indeed, coupling aggregative nesting with deep nest construction and longer incubation was found to impart positive synergistic effects on hatching success, the significance of which was an order of magnitude greater than either strategy acting alone. Moreover, while nest excavations revealed that ovicide *does* occur rarely in large aggregations, secondary intruders generally avoided existing egg clutches, instead digging isolated egg chambers branching off of shared tunnels (Figure 3c). In fact, some properties inherent to communal nest sites appeared to introduce unique *benefits* to incubating clutches. Consistent with reports from a number of studies of nesting *Cyclura* (Iverson et al., 2004; Wiewandt, 1993), we noted old eggshells within freshly excavated nests, suggesting that tunnel networks are frequently used for more than one season. Repetitive use of tunnels is consistent with our hypothesis that communal nesting reduces energetic excavation costs. In addition, it suggests that iguanas regularly and reliably use social cues as indicators of site quality.

## 5 CONCLUSIONS

Our study has characterized flexibility in nesting strategies expressed by a free‐ranging, long‐lived reptile and identified individual‐level factors associated with the preference and performance of these strategies. These findings contribute to an expanded understanding of the trade‐offs that maintain alternative female reproductive tactics in nature, particularly in systems for which selection is concentrated at the time of nesting and for which postnatal parental care is absent. We show that while nesting in high‐density areas increases the vulnerability of females to exploitation and ovicide by later conspecifics, rock iguanas may be capable of postponing reproductive efforts for more optimal competitive conditions without delaying the development of their offspring. This can be accomplished by modifying the structure of subterranean nests to promote shorter incubation times. Further, we show that larger females appear more capable than smaller females of investing in extended nest defense and that nesting in high‐density areas can actually lead to higher reproductive success if it is coupled with factors such as deep nest excavation and long incubation times. Thus, we have illustrated in a non‐cooperative animal society how phenological and physical constraints on communal nesting strategies may be overcome by modulating through the modulation of other facets of reproductive behavior (i.e., nest construction strategies). Future studies may investigate additional parameters (e.g., substrate moisture, root densities, and vegetative cover) that explain interactions between the physical incubation environment and social environment of nests and incorporate longitudinal datasets to better understand consequences for offspring performance, growth, and survivorship.

## CONFLICT OF INTERESTS

The authors have no competing interests to declare.

## AUTHOR CONTRIBUTIONS

J.B.M. implemented the study design, directed field data collection, conducted data analysis, and was responsible for the majority of manuscript preparation. T.L. and T.O. were heavily involved with field collection, with T.L. independently directing and carrying out field collection in 2018. M.G. and G.P.G. conducted all preliminary surveys identifying active nesting areas and advised and assisted with fieldwork and manuscript preparation. M.E.W. advised and assisted with study design, data interpretation, and manuscript preparation.

## Data Availability

Raw data for all individual nests, including dates, structural dimensions, and parameters of female morphology and reproductive output, are accessible through the Dryad Digital Repository (https://doi.org/10.5061/dryad.jwstqjq5s).
