# Supplementary figures and images for: Conditional female strategies influence hatching success in a communally nesting iguana
Source: Ecol Evol. 2020 Mar 4;10(7):3424–38. doi: 10.1002/ece3.6139 (PMC7141077; doi:10.1002/ece3.6139)

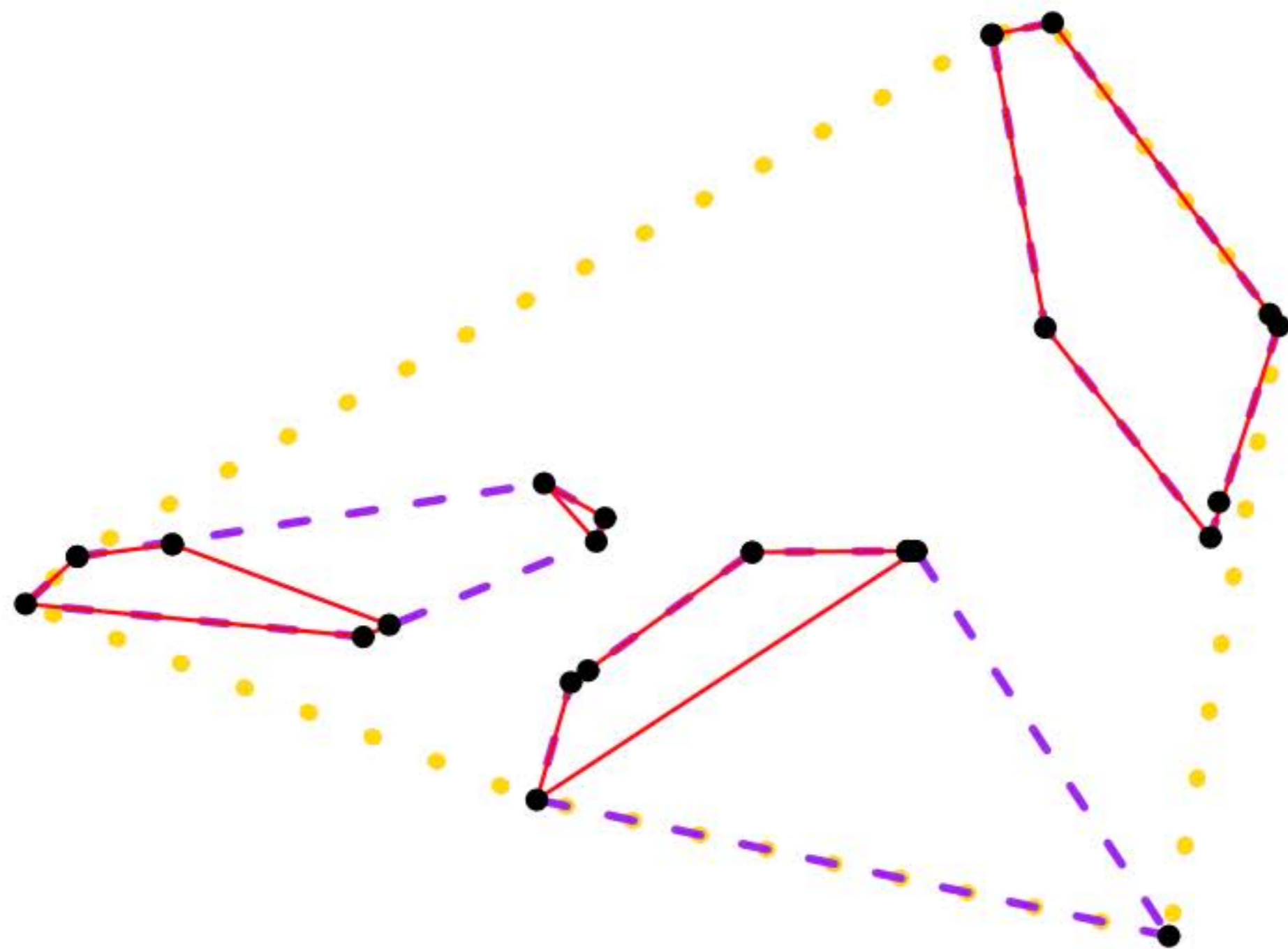

Supplement: Supplementary file 1 [file ECE3-10-3424-s001.pdf]

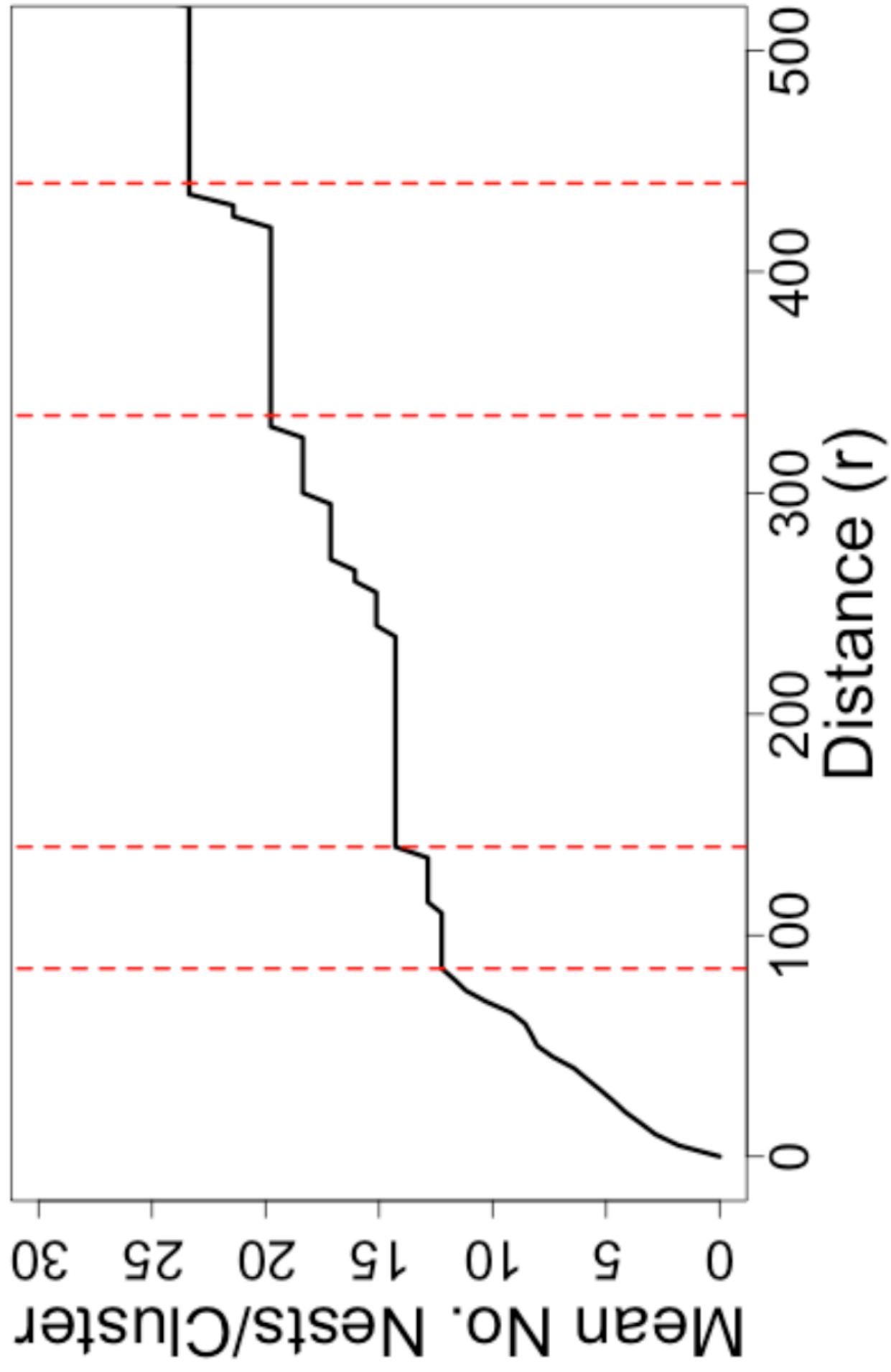

Supplement: Supplementary file 2 [file ECE3-10-3424-s002.pdf]

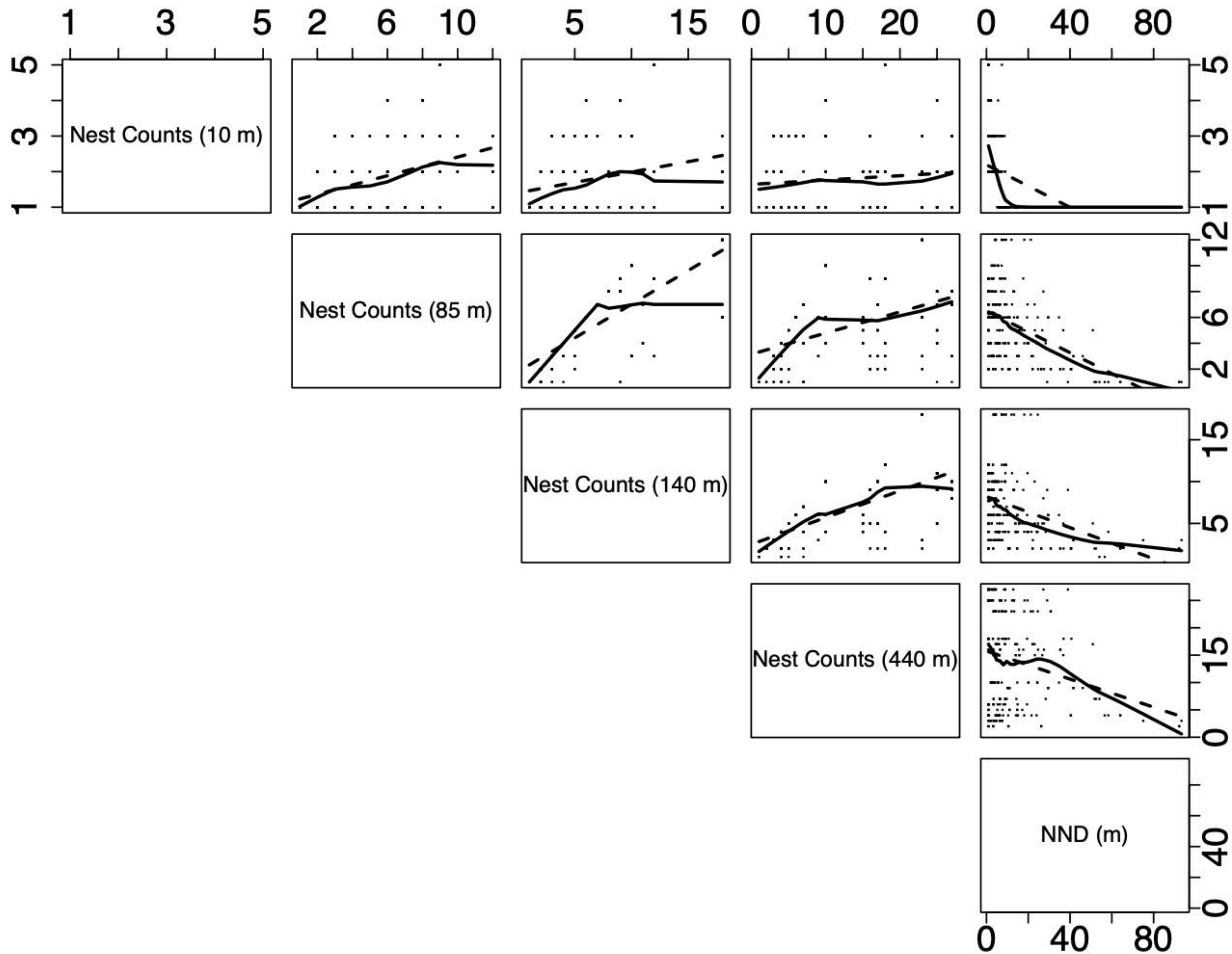

Supplement: Supplementary file 3 [file ECE3-10-3424-s003.pdf]

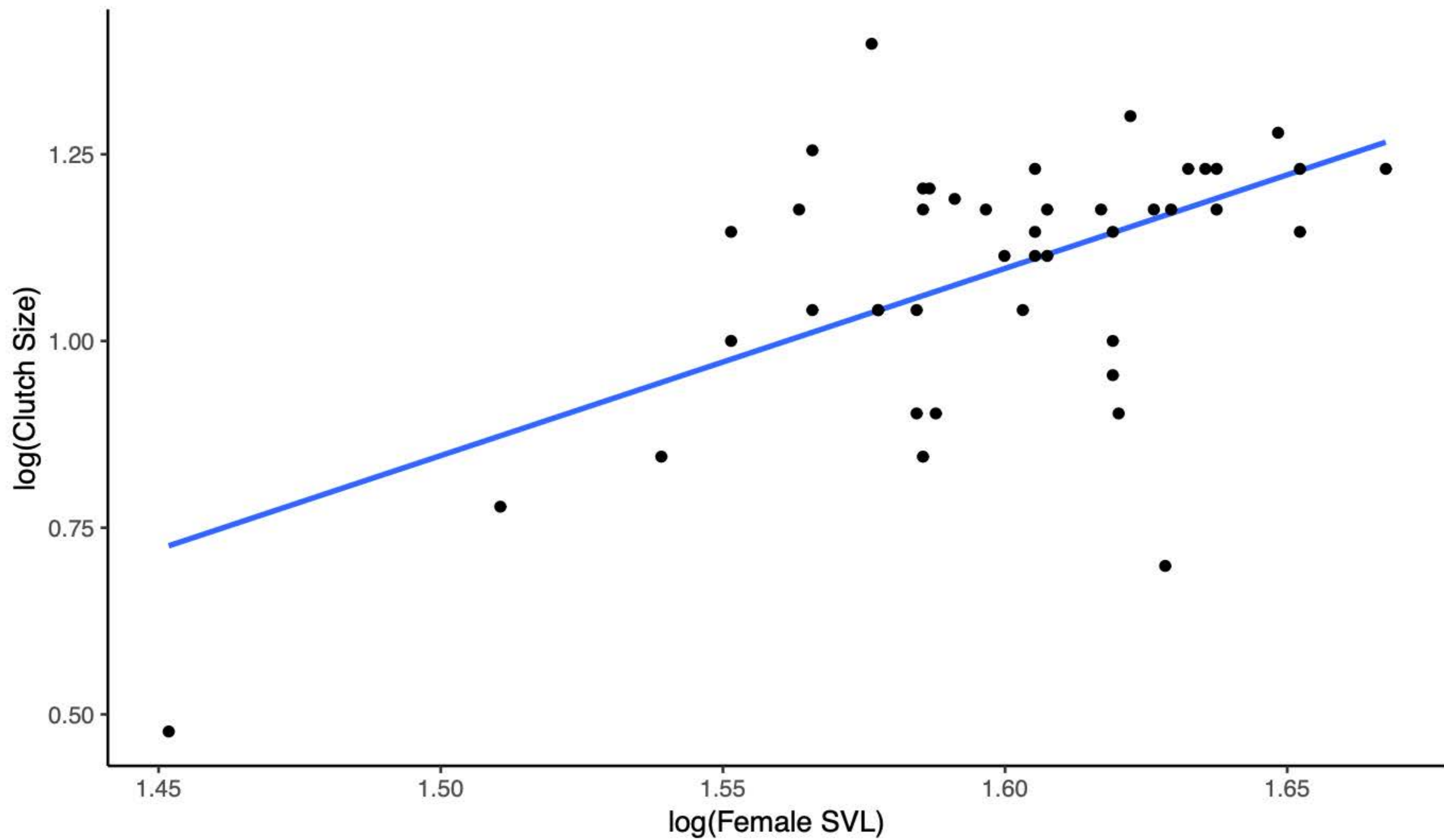

Supplement: Supplementary file 5 [file ECE3-10-3424-s005.pdf]
