# Supplementary material for: Conditional female strategies influence hatching success in a communally nesting iguana
Source: Ecol Evol. 2020 Mar 4;10(7):3424–38. doi: 10.1002/ece3.6139 (PMC7141077; doi:10.1002/ece3.6139)
Supplement: Supplementary file 7 [file ECE3-10-3424-s007.docx]

**APPENDIX**

Appendix S1: Surveys in 2010 versus 2015-18

In 2010, M. Goetz led a team of scientists from Durrell Wildlife Conservation Trust to complete nesting surveys over the whole coastline of Little Cayman, with the objective of identifying hot-spots for iguana nesting that should receive priority protection. This consisted of teams of 2–3 people surveying all areas of the 32-km coastline that 1) contained suitable substrate for digging and 2) were not completely shaded, at least twice per week. Sections were walked systematically and nests were marked with colored flags to prevent repeat counts. In contrast, investigations between 2015 and 2018 were chiefly intended to characterize individual variation in nesting strategies. Thus, surveys targeted select sites and were conducted on a more regular basis (twice per day). This intensive monitoring facilitated clear differentiation between trial digs, complete nests, and nests that were re-opened and subsequently re-sealed. Return visits to sites throughout the hatching season served as a secondary check to confirm presence of egg chambers and to detect nests that escaped notice during initial construction. In the former approach, overestimation was possible due to the regularity with which excavations resembling iguana nests are incidentally formed in soft substrate. These may be attributed to weathering or incomplete trial digs. In the latter approach, underestimation is possible in cryptic circumstances where females and hatchlings go undetected, and when excavations fail to locate egg chambers. For consistency, only data collected during 2015–2018 surveys is presented here.

Appendix S2: Surveyed nesting sites (as numbered in Fig 2)

(1) Bloody Bay: a small coastal site containing soft, sandy substrate and shrubby vegetation. Supported 4-6 nests each survey year between 2015 and 2017.

(2) Phosphate Mound: a large mound of earth artificially deposited many decades ago along a forested walking trail through the west end interior. Supported 3–5 nests each survey year between 2015 and 2017.

(3) Northwest: a small coastal site containing rocky substrate and mixed high-canopy and shrubby vegetation. Supported 4–6 nests each survey year between 2015 and 2017.

(4) Lighthouse: a medium-sized coastal site located at the westernmost tip of the island and containing sandy, soft substrate and patchy high-canopy vegetation. Supported 3–5 nests each survey year between 2015 and 2017.

(5) Preston Bay: the largest communal site located on the southwestern coastline containing pristine coastal shrubland bordered by mangrove. The Little Cayman National Trust protected a large portion of the site in 2013. Supported 20–30 nests each survey year between 2015 and 2017.

(6) Airport: a recently cleared (~15 years ago) large coastal site containing soft, sandy substrate and dominated by early successive shrubs and grasses. Supported 8–10 nests each survey year between 2015 and 2017.

(7) Spot Bay: a small outcropping in dry shrubland off a major cross-island road. Contains a few small deposits of rocky but dig-able substrate. Supported 2–3 nests each survey year between 2015 and 2017.

(8) Museum: a large human-modified site located immediately adjacent to the town square. Contains several buildings and little natural vegetation, but soft sand is plentiful and very deep. Supports a large (~50) resident population of iguanas and supported 13–15 nests each survey year between 2015 and 2017.

Appendix S3: Notes on inclement weather

Heavy precipitation impeded our nest surveys on a number of days each year (2015: Jun 2–5; 2016: Jun 3–6; 2017: Jun 2 and Jun 18–19; 2018: May 18–28, Jun 16, Jun 24); however, it is unlikely that such weather conditions permitted nesting activity.

Appendix S4: Nest excavations

To minimize the risk of egg desiccation, excavations were carried out only in the early morning (0500–0900) or the early evening (1600–1900). Ideally within one week of a nest being closed (to avoid compaction), the soft substrate that was used to fill the entry tunnel was dug out by hand and the tunnel was followed to its egg chamber. Because many nests remained active with the intrusion of conspecifics in the days and weeks following initial closure, excavations were occasionally delayed for up to four weeks until activity subsided. Egg chambers could be oriented at the terminus of entry tunnels or could “bud” laterally off of extending tunnels. Incubating eggs were delicately removed from egg chambers without rotating, marked externally with black marker, measured with calipers, and weighed in plastic bags with 50–100 g spring scales (Pesola®, Feusisberg, Switzerland). The number of viable and inviable eggs was documented at the start of incubation to project final clutch hatching success. All eggs were returned to their original position and the chamber was re-sealed with compacted substrate. We then used wooden folding rulers to measure the length of each tunnel in its entirety (to the final point of substrate compaction) and chamber depth from chamber floor to the soil surface at the mouth of the egg chamber.

Appendix S5: Edge-thinning

Edge-thinning is a statistical method of spatial cluster detection that requires that the distance between points in a cluster not be greater than some threshold distance. In this way, detected clusters are hierarchically nested (Figure S1). Looking across a range of threshold distances allows users to identify plateaus in the mean number of points within a cluster, which represent hierarchical scales of organization. In the plot below, I visually identified threshold distances for clustering at 85 m, 140 m, 340 m, and 440 m (Figure S2).

Figure S1: Illustration of hierarchical clustering of nests at Preston Bay in 2015. Clusters occur at three threshold distances: 85 m, 140, and 440 m.

Figure S2: Visualization of plateaus in mean number of nests across a range of distances. Red dotted lines are drawn at threshold distances: 85 m, 140 m, 340 m, and 440 m.

Figure S3: Pairwise correlations between various measures of local nest densities: Number of neighbors measured at four threshold distances (10 m, 85 m, 140 m, and 440 m) and nearest neighbor distance (NND).

Figure S4: Phenological patterns of nest closures and emergences by year. Blue lines represent observed nest closures and green lines represent observed nest emergences. Survey dates are bounded within nonshaded areas, and 15% and 85% quantiles of nesting activity each year are bounded by red dashed lines.

Figure S5: Log-log regression of female SVL and clutch size.

Figure S6: Predicted relationship between incubation duration and nest start date, overlaying observed data points.
